# {2,2′-[(5-Bromo­pyridine-2,3-di­yl)bis­(nitrilo­methyl­idyne)]diphenolato}chlorido(*N*,*N*-dimethyl­formamide)iron(III)

**DOI:** 10.1107/S1600536809031432

**Published:** 2009-09-12

**Authors:** Ning Sheng, Zhonghai Ni

**Affiliations:** a Department of Chemistry & Chemical Engineering, Jining University, Qufu 273155, People’s Republic of China; b School of Chemistry & Chemical Engineering, Shandong University, Jinan 250100, People’s Republic of China

## Abstract

In the title complex, [Fe(C_19_H_12_BrClN_3_O_2_)(C_3_H_7_NO)], the Fe^III^ atom is coordinated by an *N*,*N*,*O*,*O*-tetra­dentate Schiff base ligand and *trans* coordinated by a chloride anion and the O atom of an *N*,*N*-dimethyl­formamide mol­ecule. The resulting geometry is distorted octa­hedral within a ClN_2_O_3_ donor set.

## Related literature

For the optical, electronic, magnetic, biological and catalytic properties of complexes containing salicylaldehyde ligands, see: Alam *et al.* (2003 ▶); Oshiob *et al.* (2005 ▶); Zelewsky & von Knof (1999 ▶).
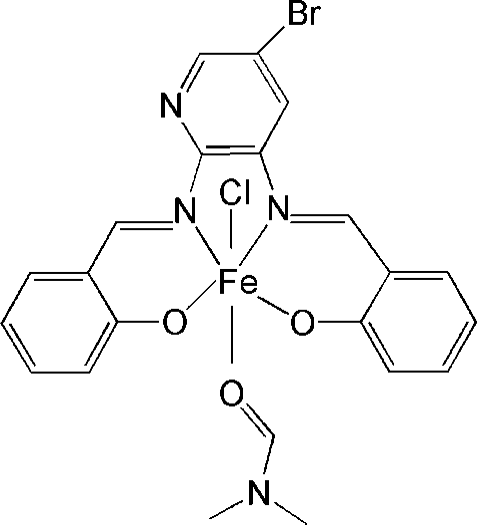


## Experimental

### 

#### Crystal data

[Fe(C_19_H_12_BrClN_3_O_2_)(C_3_H_7_NO)]
*M* *_r_* = 558.62
Monoclinic, 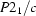

*a* = 13.1626 (11) Å
*b* = 15.3553 (13) Å
*c* = 12.6376 (11) Å
β = 118.186 (1)°
*V* = 2251.4 (3) Å^3^
*Z* = 4
Mo *K*α radiation
μ = 2.60 mm^−1^
*T* = 293 K
0.21 × 0.15 × 0.11 mm

#### Data collection

Bruker APEXII CCD area-detector diffractometer
Absorption correction: multi-scan (*SADABS*; Sheldrick, 1996 ▶) *T*_min_ = 0.612, *T*_max_ = 0.763
11869 measured reflections
4421 independent reflections
3468 reflections with *I* > 2σ(*I*)
*R*_int_ = 0.023

#### Refinement

*R*[*F* ^2^ > 2σ(*F* ^2^)] = 0.039
*wR*(*F* ^2^) = 0.117
*S* = 1.04
4421 reflections
291 parameters
H-atom parameters constrained
Δρ_max_ = 1.35 e Å^−3^
Δρ_min_ = −0.39 e Å^−3^

### 

Data collection: *APEX2* (Bruker, 2004 ▶); cell refinement: *SAINT-Plus* (Bruker, 2004 ▶); data reduction: *SAINT-Plus*; program(s) used to solve structure: *SHELXS97* (Sheldrick, 2008 ▶); program(s) used to refine structure: *SHELXL97* (Sheldrick, 2008 ▶); molecular graphics: *SHELXL97*; software used to prepare material for publication: *SHELXL97*.

## Supplementary Material

Crystal structure: contains datablocks global, I. DOI: 10.1107/S1600536809031432/tk2520sup1.cif

Structure factors: contains datablocks I. DOI: 10.1107/S1600536809031432/tk2520Isup2.hkl

Additional supplementary materials:  crystallographic information; 3D view; checkCIF report

## Figures and Tables

**Fig. 1.**
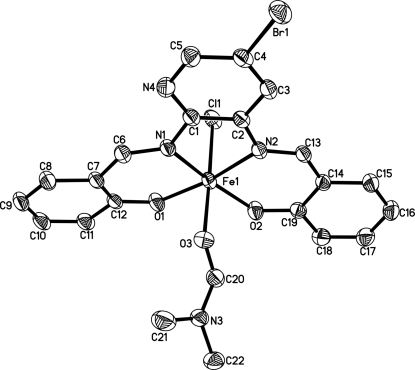
The molecular structure of (I) showing the atom-labelling scheme. Displacement ellipsoids are drawn at the 30% probability level. Hydrogen atoms have been omitted for clarity.

## supplementary crystallographic information

### Comment

The synthesis of complexes containing salicylaldehyde ligands has attracted continuous research interest not only because of their appealing structural and topological novelty, but also due to their unusual optical, electronic, magnetic, biological, and catalytic properties (Alam *et al.*, 2003; Zelewsky *et al.*, 1999; Oshiob *et al.*, 2005). In the present paper, we describe the synthesis and structural characterization of the title compound, (I).

In (I), the Fe^III^ atom is tetracoordinated by Schiff base ligand *via* two N and two O atoms, Fig. 1. In addition the metal centre is coordinated by a Cl anion and the O atom of a *N*,*N*-dimethylformamide molecule. The resulting coordination geometry is based on a distorted octahedron in which the Cl and *N*,*N*-dimethylformamide-O atoms define axial sites.

### Experimental

Condensation of 4-bromo-*o*-phenylenediamine with salicylaldehyde in a 1:2 molar ratio in ethanol gave the Schiff base ligand. FeCl_3_ (0.1 mmol) was added dropwise to a solution of the Schiff base (0.1 mmol) in methanol. The resulting solution was stirred at room temperature for 30 minutes. After filtering, the insoluble solids were dissolved in DMF and ether. The product was isolated red-brown crystals in a yield of 45% after a few weeks.

### Refinement

Hydrogen atoms were placed at calculated positions (C–H 0.93–0.96 Å) and were treated as riding on their parent atoms with *U*(H) set to 1.2*U*_eq_(C). The maximum and minimum residual electron density peaks of 1.35 and 0.39 eÅ^-3^, respectively, were located 1.07 Å and 0.69 Å from the H5 and Br1 atoms, respectively.

### Crystal data

**Table:**

[Fe(C_19_H_12_BrClN_3_O_2_)(C_3_H_7_NO)]	*Z* = 4
*M**_r_* = 558.62	*F*(000) = 1122
Monoclinic, *P*2_1_/*c*	*D*_x_ = 1.647 Mg m^−^^3^
Hall symbol: -P 2ybc	Mo *K*α radiation, λ = 0.71073 Å
*a* = 13.1626 (11) Å	µ = 2.60 mm^−^^1^
*b* = 15.3553 (13) Å	*T* = 293 K
*c* = 12.6376 (11) Å	Block, red-brown
β = 118.186 (1)°	0.21 × 0.15 × 0.11 mm
*V* = 2251.4 (3) Å^3^	

### Data collection

**Table:**

Bruker APEXII CCD area-detector diffractometer	4421 independent reflections
Radiation source: fine-focus sealed tube	3468 reflections with *I* > 2σ(*I*)
graphite	*R*_int_ = 0.023
φ and ω scans	θ_max_ = 26.0°, θ_min_ = 2.2°
Absorption correction: multi-scan (*SADABS*; Sheldrick, 1996)	*h* = −16→16
*T*_min_ = 0.612, *T*_max_ = 0.763	*k* = −14→18
11869 measured reflections	*l* = −15→13

### Refinement

**Table:**

Refinement on *F*^2^	Primary atom site location: structure-invariant direct methods
Least-squares matrix: full	Secondary atom site location: difference Fourier map
*R*[*F*^2^ > 2σ(*F*^2^)] = 0.039	Hydrogen site location: inferred from neighbouring sites
*wR*(*F*^2^) = 0.117	H-atom parameters constrained
*S* = 1.04	*w* = 1/[σ^2^(*F*_o_^2^) + (0.0664*P*)^2^ + 1.0974*P*] where *P* = (*F*_o_^2^ + 2*F*_c_^2^)/3
4421 reflections	(Δ/σ)_max_ = 0.002
291 parameters	Δρ_max_ = 1.35 e Å^−^^3^
0 restraints	Δρ_min_ = −0.39 e Å^−^^3^

### Special details

**Table:**

Geometry. All e.s.d.'s (except the e.s.d. in the dihedral angle between two l.s. planes) are estimated using the full covariance matrix. The cell e.s.d.'s are taken into account individually in the estimation of e.s.d.'s in distances, angles and torsion angles; correlations between e.s.d.'s in cell parameters are only used when they are defined by crystal symmetry. An approximate (isotropic) treatment of cell e.s.d.'s is used for estimating e.s.d.'s involving l.s. planes.
Refinement. Refinement of *F*^2^ against ALL reflections. The weighted *R*-factor *wR* and goodness of fit *S* are based on *F*^2^, conventional *R*-factors *R* are based on *F*, with *F* set to zero for negative *F*^2^. The threshold expression of *F*^2^ > σ(*F*^2^) is used only for calculating *R*-factors(gt) *etc*. and is not relevant to the choice of reflections for refinement. *R*-factors based on *F*^2^ are statistically about twice as large as those based on *F*, and *R*- factors based on ALL data will be even larger.

### Fractional atomic coordinates and isotropic or equivalent isotropic displacement parameters (Å^2^)

**Table:**

	*x*	*y*	*z*	*U*_iso_*/*U*_eq_	
Fe1	0.81172 (4)	0.45734 (3)	0.12689 (4)	0.04193 (15)	
Br1	0.45611 (3)	0.63148 (3)	0.36373 (4)	0.06309 (16)	
Cl1	0.68099 (7)	0.34010 (6)	0.05361 (9)	0.0584 (3)	
O1	0.94591 (19)	0.38509 (16)	0.1821 (2)	0.0511 (6)	
O2	0.78233 (19)	0.49869 (16)	−0.0266 (2)	0.0505 (6)	
O3	0.9319 (2)	0.56438 (16)	0.2007 (2)	0.0555 (6)	
N1	0.8280 (2)	0.45132 (16)	0.3011 (2)	0.0412 (6)	
N2	0.6755 (2)	0.54061 (16)	0.1157 (2)	0.0401 (6)	
N3	1.0807 (2)	0.63665 (18)	0.1998 (3)	0.0498 (7)	
N4	0.7329 (2)	0.48025 (19)	0.4177 (3)	0.0501 (7)	
C1	0.7382 (3)	0.4908 (2)	0.3158 (3)	0.0410 (7)	
C2	0.6585 (3)	0.53962 (19)	0.2178 (3)	0.0394 (7)	
C3	0.5712 (3)	0.5822 (2)	0.2311 (3)	0.0450 (7)	
H3	0.5163	0.6158	0.1694	0.054*	
C4	0.5694 (3)	0.5727 (2)	0.3380 (3)	0.0472 (8)	
C5	0.6484 (3)	0.5206 (2)	0.4282 (3)	0.0515 (8)	
H5	0.6426	0.5135	0.4982	0.062*	
C6	0.9158 (3)	0.4183 (2)	0.3952 (3)	0.0452 (7)	
H6	0.9172	0.4251	0.4690	0.054*	
C7	1.0095 (3)	0.3728 (2)	0.3937 (3)	0.0454 (8)	
C8	1.0937 (3)	0.3383 (2)	0.5057 (3)	0.0551 (9)	
H8	1.0876	0.3493	0.5748	0.066*	
C9	1.1837 (3)	0.2891 (3)	0.5130 (4)	0.0632 (11)	
H9	1.2383	0.2667	0.5864	0.076*	
C10	1.1924 (3)	0.2731 (2)	0.4092 (4)	0.0612 (10)	
H10	1.2529	0.2392	0.4141	0.073*	
C11	1.1146 (3)	0.3058 (2)	0.3007 (4)	0.0547 (9)	
H11	1.1235	0.2947	0.2332	0.066*	
C12	1.0196 (3)	0.3567 (2)	0.2898 (3)	0.0448 (8)	
C13	0.6053 (3)	0.5821 (2)	0.0183 (3)	0.0414 (7)	
H13	0.5427	0.6095	0.0191	0.050*	
C14	0.6146 (3)	0.5897 (2)	−0.0883 (3)	0.0424 (7)	
C15	0.5322 (3)	0.6421 (2)	−0.1808 (3)	0.0524 (9)	
H15	0.4718	0.6658	−0.1716	0.063*	
C16	0.5389 (3)	0.6588 (3)	−0.2836 (3)	0.0594 (10)	
H16	0.4838	0.6936	−0.3434	0.071*	
C17	0.6287 (3)	0.6233 (3)	−0.2976 (4)	0.0604 (10)	
H17	0.6344	0.6355	−0.3667	0.072*	
C18	0.7094 (3)	0.5704 (2)	−0.2111 (3)	0.0539 (9)	
H18	0.7687	0.5475	−0.2229	0.065*	
C19	0.7045 (3)	0.5504 (2)	−0.1065 (3)	0.0439 (7)	
C20	0.9823 (3)	0.5955 (2)	0.1477 (3)	0.0512 (8)	
H20	0.9474	0.5890	0.0647	0.061*	
C21	1.1401 (4)	0.6472 (3)	0.3283 (4)	0.0786 (13)	
H21A	1.1111	0.6978	0.3498	0.118*	
H21B	1.2212	0.6541	0.3553	0.118*	
H21C	1.1277	0.5967	0.3654	0.118*	
C22	1.1359 (3)	0.6718 (3)	0.1329 (4)	0.0615 (10)	
H22A	1.0840	0.6676	0.0484	0.092*	
H22B	1.2047	0.6394	0.1519	0.092*	
H22C	1.1553	0.7318	0.1542	0.092*	

### Atomic displacement parameters (Å^2^)

**Table:**

	*U*^11^	*U*^22^	*U*^33^	*U*^12^	*U*^13^	*U*^23^
Fe1	0.0323 (2)	0.0442 (3)	0.0446 (3)	0.00752 (19)	0.0144 (2)	0.0030 (2)
Br1	0.0530 (2)	0.0760 (3)	0.0671 (3)	0.01648 (18)	0.0340 (2)	0.00264 (19)
Cl1	0.0388 (4)	0.0490 (5)	0.0738 (6)	0.0018 (4)	0.0156 (4)	−0.0049 (4)
O1	0.0363 (12)	0.0610 (15)	0.0522 (14)	0.0125 (10)	0.0177 (11)	0.0032 (11)
O2	0.0456 (13)	0.0555 (14)	0.0484 (13)	0.0124 (11)	0.0205 (11)	0.0053 (11)
O3	0.0466 (13)	0.0568 (15)	0.0615 (15)	−0.0083 (11)	0.0242 (12)	−0.0003 (12)
N1	0.0337 (13)	0.0393 (14)	0.0467 (15)	0.0040 (11)	0.0158 (12)	0.0030 (11)
N2	0.0331 (13)	0.0386 (14)	0.0421 (14)	0.0028 (11)	0.0124 (11)	0.0006 (11)
N3	0.0424 (15)	0.0472 (16)	0.0608 (19)	0.0015 (12)	0.0252 (14)	0.0027 (13)
N4	0.0489 (16)	0.0571 (17)	0.0465 (16)	0.0089 (14)	0.0244 (14)	0.0070 (13)
C1	0.0333 (16)	0.0375 (16)	0.0491 (18)	0.0010 (13)	0.0169 (14)	−0.0013 (13)
C2	0.0326 (15)	0.0386 (16)	0.0434 (17)	−0.0005 (12)	0.0151 (13)	−0.0031 (13)
C3	0.0350 (16)	0.0421 (17)	0.0494 (19)	0.0038 (13)	0.0131 (14)	0.0007 (14)
C4	0.0375 (17)	0.0488 (19)	0.057 (2)	0.0019 (14)	0.0239 (16)	−0.0022 (16)
C5	0.053 (2)	0.055 (2)	0.051 (2)	0.0086 (17)	0.0283 (17)	0.0060 (16)
C6	0.0380 (17)	0.0472 (18)	0.0455 (19)	0.0030 (14)	0.0157 (15)	0.0015 (14)
C7	0.0305 (16)	0.0424 (18)	0.054 (2)	0.0017 (13)	0.0119 (14)	0.0072 (14)
C8	0.0396 (18)	0.056 (2)	0.055 (2)	0.0002 (16)	0.0102 (16)	0.0072 (17)
C9	0.0354 (18)	0.057 (2)	0.074 (3)	0.0052 (16)	0.0073 (18)	0.019 (2)
C10	0.0312 (17)	0.051 (2)	0.090 (3)	0.0099 (15)	0.0195 (19)	0.0130 (19)
C11	0.0398 (18)	0.0478 (19)	0.076 (3)	0.0064 (15)	0.0266 (18)	0.0050 (17)
C12	0.0274 (15)	0.0389 (16)	0.060 (2)	0.0006 (12)	0.0140 (15)	0.0058 (14)
C13	0.0313 (15)	0.0369 (16)	0.0504 (19)	0.0021 (13)	0.0145 (14)	0.0007 (14)
C14	0.0333 (15)	0.0376 (16)	0.0442 (18)	−0.0038 (13)	0.0084 (14)	0.0019 (13)
C15	0.0421 (19)	0.053 (2)	0.052 (2)	0.0020 (15)	0.0136 (16)	0.0062 (16)
C16	0.054 (2)	0.059 (2)	0.051 (2)	0.0041 (18)	0.0129 (18)	0.0119 (17)
C17	0.061 (2)	0.068 (2)	0.046 (2)	−0.0066 (19)	0.0198 (18)	0.0068 (17)
C18	0.052 (2)	0.059 (2)	0.052 (2)	−0.0045 (17)	0.0257 (18)	−0.0023 (17)
C19	0.0378 (17)	0.0435 (18)	0.0429 (18)	−0.0063 (13)	0.0128 (14)	−0.0031 (14)
C20	0.0449 (19)	0.051 (2)	0.053 (2)	−0.0002 (16)	0.0191 (17)	−0.0008 (16)
C21	0.061 (3)	0.101 (3)	0.064 (3)	−0.027 (2)	0.022 (2)	−0.004 (2)
C22	0.056 (2)	0.062 (2)	0.080 (3)	0.0015 (18)	0.043 (2)	0.008 (2)

### Geometric parameters (Å, °)

**Table:**

Fe1—O2	1.900 (2)	C7—C8	1.424 (5)
Fe1—O1	1.917 (2)	C8—C9	1.371 (5)
Fe1—N1	2.110 (3)	C8—H8	0.9300
Fe1—N2	2.152 (3)	C9—C10	1.391 (6)
Fe1—O3	2.164 (2)	C9—H9	0.9300
Fe1—Cl1	2.3566 (10)	C10—C11	1.361 (5)
Br1—C4	1.899 (3)	C10—H10	0.9300
O1—C12	1.317 (4)	C11—C12	1.425 (5)
O2—C19	1.312 (4)	C11—H11	0.9300
O3—C20	1.238 (4)	C13—C14	1.414 (5)
N1—C6	1.307 (4)	C13—H13	0.9300
N1—C1	1.416 (4)	C14—C15	1.412 (5)
N2—C13	1.304 (4)	C14—C19	1.441 (5)
N2—C2	1.409 (4)	C15—C16	1.367 (5)
N3—C20	1.306 (4)	C15—H15	0.9300
N3—C21	1.441 (5)	C16—C17	1.388 (6)
N3—C22	1.454 (5)	C16—H16	0.9300
N4—C1	1.332 (4)	C17—C18	1.373 (5)
N4—C5	1.334 (4)	C17—H17	0.9300
C1—C2	1.403 (4)	C18—C19	1.388 (5)
C2—C3	1.399 (4)	C18—H18	0.9300
C3—C4	1.370 (5)	C20—H20	0.9300
C3—H3	0.9300	C21—H21A	0.9600
C4—C5	1.378 (5)	C21—H21B	0.9600
C5—H5	0.9300	C21—H21C	0.9600
C6—C7	1.426 (5)	C22—H22A	0.9600
C6—H6	0.9300	C22—H22B	0.9600
C7—C12	1.404 (5)	C22—H22C	0.9600
			
O2—Fe1—O1	105.85 (10)	C9—C8—H8	119.5
O2—Fe1—N1	162.06 (10)	C7—C8—H8	119.5
O1—Fe1—N1	88.45 (10)	C8—C9—C10	119.1 (3)
O2—Fe1—N2	88.45 (10)	C8—C9—H9	120.5
O1—Fe1—N2	164.57 (11)	C10—C9—H9	120.5
N1—Fe1—N2	76.44 (10)	C11—C10—C9	121.9 (3)
O2—Fe1—O3	86.61 (10)	C11—C10—H10	119.1
O1—Fe1—O3	85.54 (10)	C9—C10—H10	119.1
N1—Fe1—O3	83.71 (10)	C10—C11—C12	120.5 (4)
N2—Fe1—O3	89.66 (10)	C10—C11—H11	119.7
O2—Fe1—Cl1	95.47 (8)	C12—C11—H11	119.7
O1—Fe1—Cl1	94.43 (8)	O1—C12—C7	124.0 (3)
N1—Fe1—Cl1	94.13 (8)	O1—C12—C11	117.8 (3)
N2—Fe1—Cl1	89.83 (7)	C7—C12—C11	118.2 (3)
O3—Fe1—Cl1	177.85 (8)	N2—C13—C14	126.8 (3)
C12—O1—Fe1	132.1 (2)	N2—C13—H13	116.6
C19—O2—Fe1	134.1 (2)	C14—C13—H13	116.6
C20—O3—Fe1	121.9 (2)	C15—C14—C13	117.3 (3)
C6—N1—C1	118.7 (3)	C15—C14—C19	118.3 (3)
C6—N1—Fe1	125.2 (2)	C13—C14—C19	124.4 (3)
C1—N1—Fe1	116.0 (2)	C16—C15—C14	121.7 (4)
C13—N2—C2	121.4 (3)	C16—C15—H15	119.2
C13—N2—Fe1	123.3 (2)	C14—C15—H15	119.2
C2—N2—Fe1	114.89 (19)	C15—C16—C17	119.3 (4)
C20—N3—C21	120.2 (3)	C15—C16—H16	120.4
C20—N3—C22	122.4 (3)	C17—C16—H16	120.4
C21—N3—C22	117.3 (3)	C18—C17—C16	121.1 (4)
C1—N4—C5	117.8 (3)	C18—C17—H17	119.5
N4—C1—C2	124.0 (3)	C16—C17—H17	119.5
N4—C1—N1	120.2 (3)	C17—C18—C19	121.5 (4)
C2—C1—N1	115.8 (3)	C17—C18—H18	119.2
C3—C2—C1	117.2 (3)	C19—C18—H18	119.2
C3—C2—N2	126.8 (3)	O2—C19—C18	119.8 (3)
C1—C2—N2	116.0 (3)	O2—C19—C14	122.1 (3)
C4—C3—C2	117.8 (3)	C18—C19—C14	118.1 (3)
C4—C3—H3	121.1	O3—C20—N3	124.8 (4)
C2—C3—H3	121.1	O3—C20—H20	117.6
C3—C4—C5	121.3 (3)	N3—C20—H20	117.6
C3—C4—Br1	120.0 (3)	N3—C21—H21A	109.5
C5—C4—Br1	118.7 (3)	N3—C21—H21B	109.5
N4—C5—C4	121.8 (3)	H21A—C21—H21B	109.5
N4—C5—H5	119.1	N3—C21—H21C	109.5
C4—C5—H5	119.1	H21A—C21—H21C	109.5
N1—C6—C7	125.2 (3)	H21B—C21—H21C	109.5
N1—C6—H6	117.4	N3—C22—H22A	109.5
C7—C6—H6	117.4	N3—C22—H22B	109.5
C12—C7—C8	119.4 (3)	H22A—C22—H22B	109.5
C12—C7—C6	124.3 (3)	N3—C22—H22C	109.5
C8—C7—C6	116.2 (3)	H22A—C22—H22C	109.5
C9—C8—C7	120.9 (4)	H22B—C22—H22C	109.5

## References

[bb1] Alam, M. A., Nethaji, M. & Ray, M. (2003). *Angew. Chem. Int. Ed.* **42**, 1940–1942. 10.1002/anie.200250591 12730975

[bb2] Bruker (2004). *APEX2* and *SAINT*-Plus. Bruker AXS Inc., Madison, Wisconsin, USA.

[bb3] Oshiob, H., Nihei, M., Koizumi, S., Shiga, T., Nojiri, H., Nakano, M., Shirakawa, N. & Akatsu, M. (2005). *J. Am. Chem. Soc.* **127**, 4568–4569. 10.1021/ja042217p 15796510

[bb4] Sheldrick, G. M. (1996). *SADABS* University of Göttingen, Germany.

[bb5] Sheldrick, G. M. (2008). *Acta Cryst.* A**64**, 112–122. 10.1107/S0108767307043930 18156677

[bb6] Zelewsky, A. & von Knof, U. (1999). *Angew. Chem. Int. Ed.* **38**, 302–322. 10.1002/(SICI)1521-3773(19990201)38:3<302::AID-ANIE302>3.0.CO;2-G 29711653

